# Supplementary material for: Comparative transcriptome analysis on candidate genes associated with fruiting body growth and development in Lyophyllum decastes
Source: PeerJ. 2023 Oct 26;11:e16288. doi: 10.7717/peerj.16288 (PMC10613438; doi:10.7717/peerj.16288)
Supplement: Supplemental Information 1 — Figure S1 Venn diagram showing the unigenes annotated in different databases. Figure S2 Histogram of the gene ontology (GO) classification of the L. decastes unigenes. All of the unigenes were grouped into 59 sub-categories and summarized in three main GO categories: molecular function, cellular component and biological process. Figure S3 Histogram distribution of the COG functional categories of the L. decastes unigenes. All of the unigenes were categorized into 25 sub-categories (in the order of A to Z). Figure S4 Histogram distribution of the KEGG Pathway classification of the L. decastes unigenes. All of the unigenes were classified into 6 biological pathways on the basis of the KEGG database, including cellular processes, environmental information processing, genetic information processing, human diseases, metabolism and organismal systems. Figure S5 Volcano plots of DEGs in different comparisons (MF vs YF, MF vs PI, MF vs VM). The blue dots represent down-regulated genes, the red dots represent up-regulated genes. Figure S6 Gene ontology (GO) classification analysis of DEGs in different comparisons (MF vs YF, MF vs PI, MF vs VM). Figure S7 The KEGG pathway enrichment analysis of DEGs in different comparisons (MF vs FM, MF vs PI, MF vs VM). Figure S8 Relative expressive levels of cellulase regulators at different developmental stages. [file peerj-11-16288-s001.zip › Supplemental figures/Figure S4.pdf]

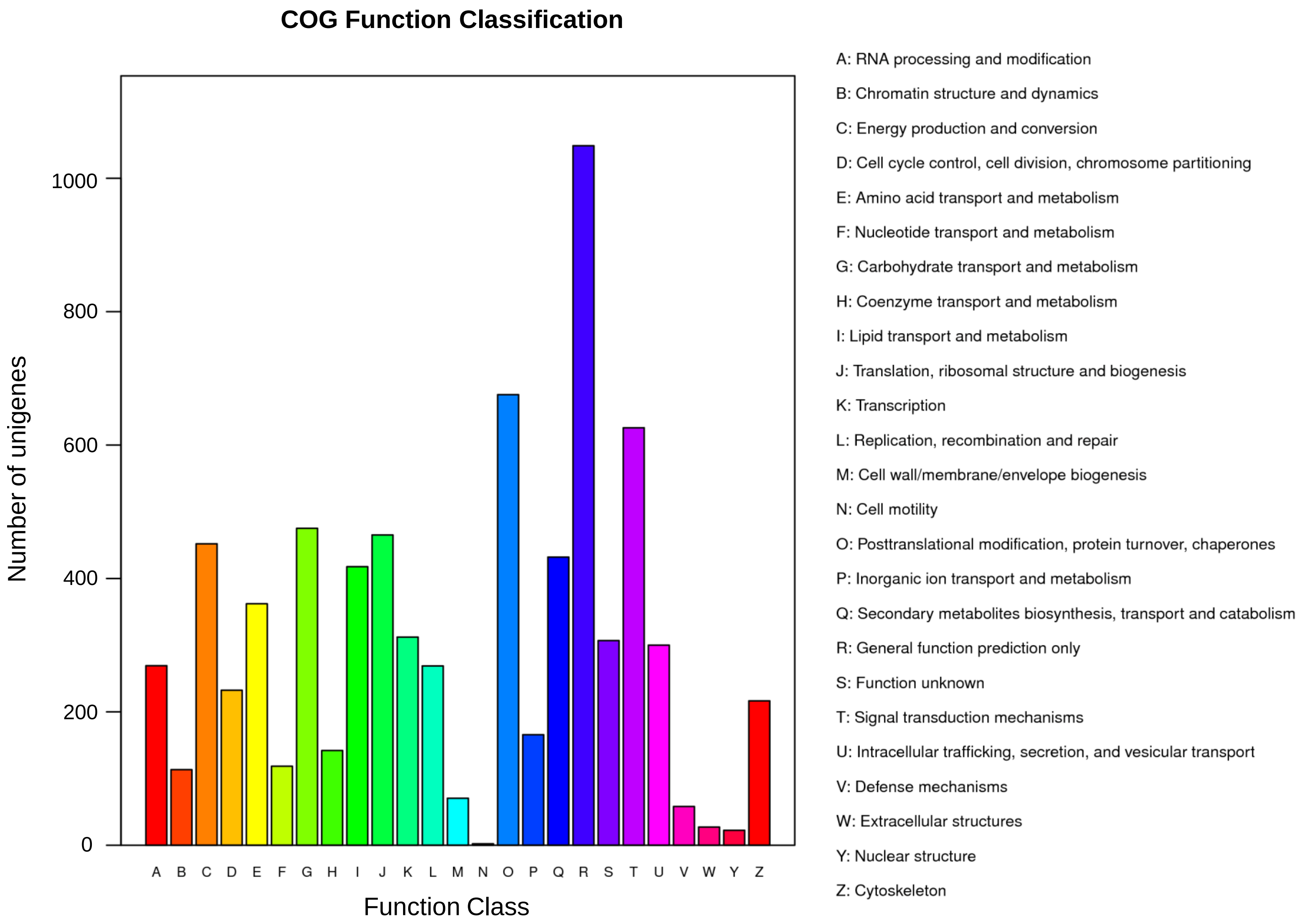
COG Function Classification
Number of unigenes
0
200
400
600
800
1000
A B C D E F G H I J K L M N O P Q R S T U V W Y Z
Function Class
A: RNA processing and modification
B: Chromatin structure and dynamics
C: Energy production and conversion
D: Cell cycle control, cell division, chromosome partitioning
E: Amino acid transport and metabolism
F: Nucleotide transport and metabolism
G: Carbohydrate transport and metabolism
H: Coenzyme transport and metabolism
I: Lipid transport and metabolism
J: Translation, ribosomal structure and biogenesis
K: Transcription
L: Replication, recombination and repair
M: Cell wall/membrane/envelope biogenesis
N: Cell motility
O: Posttranslational modification, protein turnover, chaperones
P: Inorganic ion transport and metabolism
Q: Secondary metabolites biosynthesis, transport and catabolism
R: General function prediction only
S: Function unknown
T: Signal transduction mechanisms
U: Intracellular trafficking, secretion, and vesicular transport
V: Defense mechanisms
W: Extracellular structures
Y: Nuclear structure
Z: Cytoskeleton
